# Trypanocidal Essential Oils: A Review

**DOI:** 10.3390/molecules25194568

**Published:** 2020-10-06

**Authors:** Mayara Castro de Morais, Jucieudo Virgulino de Souza, Carlos da Silva Maia Bezerra Filho, Silvio Santana Dolabella, Damião Pergentino de Sousa

**Affiliations:** 1 Laboratory of Pharmaceutical Chemistry, Department of Pharmaceutical Sciences, Federal University of Paraíba, 58051-900 João Pessoa, Paraíba, Brazil; mayaracastrodemorais@gmail.com (M.C.d.M.); vjucieudo@yahoo.com.br (J.V.d.S.); carlosmaia1996@gmail.com (C.d.S.M.B.F.); 2 Laboratory of Entomology and Tropical Parasitology, Department of Morphology, Federal University of Sergipe, 49100-000 São Cristóvão, Sergipe, Brazil; dolabellaufs@gmail.com

**Keywords:** trypanosomiasis, *Trypanosoma*, protozoan, Chagas disease, African trypanosomiasis, essential oils, natural product, terpene, neglected disease

## Abstract

Trypanosomiases are diseases caused by parasitic protozoan trypanosomes of the genus *Trypanosoma*. In humans, this includes Chagas disease and African trypanosomiasis. There are few therapeutic options, and there is low efficacy to clinical treatment. Therefore, the search for new drugs for the trypanosomiasis is urgent. This review describes studies of the trypanocidal properties of essential oils, an important group of natural products widely found in several tropical countries. Seventy-seven plants were selected from literature for the trypanocidal activity of their essential oils. The main chemical constituents and mechanisms of action are also discussed. In vitro and in vivo experimental data show the therapeutic potential of these natural products for the treatment of infections caused by species of *Trypanosoma*.

## 1. Introduction

**Figure 1:**
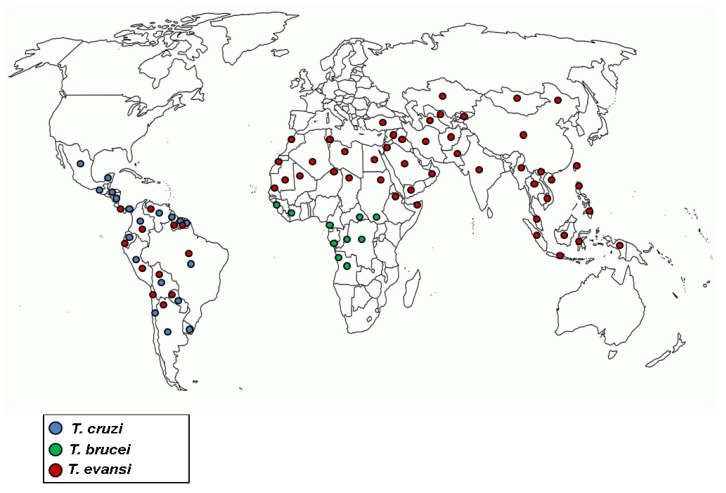
Worldwide distribution of the main species causing human trypanosomiasis.

Trypanosomiases are insect-borne parasitic diseases of humans and animals caused by flagellate protozoans of the genus *Trypanosoma*. Occurring mainly in Latin America and Africa, where they are considered endemic, and they are of great importance to human health [1,2]. According to Britannica Academic [3], the life cycle of trypanosomes includes one stage in the blood or tissue of a vertebrate host and another stage in an invertebrate vector. Furthermore, approximately 20 *Trypanosoma* species are known, but only two species cause disease in humans, *Trypanosoma cruzi* and *Trypanosoma brucei*. *T. cruzi* is the cause of American trypanosomiasis, also called Chagas disease, which is prevalent throughout the Americas. It is spread by Triatominae insects, commonly called “kissing bugs”. In the bloodstream, the parasite multiplies and can reach the heart, liver and spleen, where it can cause extensive damage. *T. brucei* is responsible for African trypanosomiasis or sleeping sickness, most commonly found in equatorial Africa. Human African trypanosomiasis takes two forms depending on the parasite involved, which are both transmitted by tsetse flies (*Glossina* spp.). Sleeping sickness in eastern and southern sub-Saharan Africa is an acute form caused by the subspecies *T. brucei rhodesiense*. Trypanosomiasis in the central and western regions of Africa is a slow-progressing form caused by *T. brucei gambiense*. Both trypanosomes invade the brain, causing mental deterioration, coma and death if left untreated [4,5]. There is also Surra, which is a trypanosomiasis caused by *T. evansi,* that affects wild and domestic animals mainly in Africa, Asia and South America, causing serious economic losses due to mortality and morbidity [6]. However, despite being a disease that predominantly affects animals, cases of surra have been reported in humans in the Asian region, where the main symptoms observed were fever and drowsiness, without invasion of the parasite in the central nervous system [7]. Figure 1 illustrates the worldwide distribution of the main species causing human trypanosomiasis.

Essential oils and their constituents present a broad spectrum of pharmacological activities, such as antinociceptive [8,9], anti-inflammatory [10], antitumor [11] and antiulcer [12] activities. The various pharmacological properties of essential oils and their constituents against *Trypanosoma* sp. are well studied [13].

The purpose of the current review is to provide a comprehensive summary of the literature on essential oils as potential trypanocidal drugs against *Trypanosoma* spp. In this study, available data could be used as an updated source of the progress or success for identification of trypanocidal compounds.

## 2. Essential Oils with Trypanocidal Activity

Forty-six articles in the literature were found that describe trypanocidal activity of essential oils, totaling seventy-seven plants studied; however, three species were not bioactive. Among trypanocidal essential oils, forty-one were active against *T. cruzi*, thirty-four against *T. brucei* and seven had activity against *T. evansi*.

The trypanocidal models/methods employed (Tables 1–3) for the in vitro and in vivo testing of trypanocidal activity of essential oils were as follows: in vitro testing with dimethyl sulfoxide (DMSO) microdilution was used for 95.1% of the oils, dissolution in 5% ethanol was used for 2.4% of the oils, and dissolution in 5% acetone was used for 2.4% of the essential oils; evaluation with colorimetric test MTT (3-(4,5-dimethylthiazol-2-yl)-2,5-diphenyltetrazolium bromide) was used for 46.7% of the oils. The resazurin test that evaluates proliferation of trypanosomes in an in vivo test was used for 6.7% of the studies.

Administration routes for parasitemia-inducing forms and blood collection for evaluation were by intraperitoneal (i.p.) and tail vein (c.d.) routes, respectively. The route of administration for essential oils was oral gavage (p.o.). In 15.5% of cases, parasitemia was evaluated through blood samples and stained using a panoptic method, while hematoxylin and eosin (HE) was used in 26.3% of cases. Scanning and transmission electron microscopy was used for 10% of cases, while measurement with a Neubauer camera was used in 25.3% of cases. ELISA was used for 15.3% of cases with an absorbance wavelength of 492–600 nm.

### 2.1. Essential Oils with Trypanocidal Activity against T. cruzi

The essential oils of the species *Cinnamodendron dinisii* Schwacker and *Siparuna guianensis* Aublet [14] presented in vitro trypanocidal activity against *T. cruzi* at concentrations of 282.93 and 209.30 µg/mL, respectively, dissolved in DMSO and analyzed using a colorimetric assay (MTT) [15,16]. The study presented by Andrade et al. [14] suggested that the trypanocidal activity of these essential oils may be associated with oxygenated terpenes found in small quantities in both plant species. Trypanocidal activity of oxygenated terpenes had already been reported in a previous study [17].

Barros et al. (2016) [18] highlighted the in vitro trypanocidal activity of the *Lantana camara* L. essential oil against *T. cruzi* at a concentration of 291.94 µg/mL dissolved in DMSO. Analysis of the essential oil of the species revealed that (*E*)-caryophyllene and bicyclogermacrene represent the principal components of this oil. Thus, (*E*)-caryophyllene may be partly responsible for the trypanocidal activity of the species. The study also highlighted that other chemical compounds in this species may present activity against *T. cruzi* [18].

The *Cinnamomum verum* J. Presl. essential oil presented in vitro anti-*T. cruzi* activity at concentrations of 24.13, 20.0 and 5.05 μg/mL and was dissolved in DMSO [19]. Inhibitory activity was assessed by Thiazolyl Blue Tetrazolium Bromide (MTT) colorimetric assay [15,16]. Analysis of the essential oils of the species revealed that (*E*)-cinnamaldehyde and eugenol were the principal constituents, suggesting that its trypanocidal activity may be related to these components. The study also pointed out that (*E*)-cinnamaldehyde was not effective against *T. cruzi* epimastigotes at low concentrations of up to 300 μM.

Essential oils from *Xylopia frutescens* var. *ferruginea* Aubl. and *Xylopia laevigata* (Mart.) [20] presented in vitro trypanocidal activity against *T. cruzi* at concentrations below 30 and 15 μg/mL, respectively. Analysis of the essential oils of both species revealed very similar chemical compositions, differing in the concentration of some constituents, yet the main components of both were bicyclogermacrene, (*E*)-caryophyllene and germacrene D. The trypanocidal activity was attributed to the high concentration of these components. Recent work has demonstrated the trypanocidal properties of these constituents [20,21].

As reported by Santoro et al. (2007) [22], the in vitro trypanocidal activity of the species *Thymus vulgaris* L. must be associated with the presence of thymol (80.4%). The author does not rule out the possibility that other components may be more active against *T. cruzi* than thymol. As a result of this study, IC_50_/24 h values equal to 77 μg/mL for epimastigotes and 38 μg/mL for trypomastigotes were found; each essential oil was dissolved in DMSO for the trypanocidal activity assays. 

The essential oil of *Aloysia triphylla* (L’Hér.) Britton [23] presented in vivo trypanocidal activity against *T. cruzi* at doses of 100 and 250 mg/kg when dissolved in DMSO and administered via an orogastric tube. Analysis of the essential oil of this species revealed citral, a mixture of two isomeric monoterpene aldehydes, geranial, and neral [24] (this last compound was the principal component, suggesting that the trypanocidal activity is related to this constituent). In a previous study, Santoro et al. (2007) [17] reported trypanocidal activity attributed to this compound when investigating the in vitro trypanocidal activity of the essential oil of *Cymbopogon citratus* (DC.) Stapf against *T. cruzi* at concentrations of 126.5 and 15.5 µg/mL. 

The *Achillea millefolium* L., *Syzygium aromaticum* L., and *Ocimum basilicum* L. essential oils [25] presented in vitro anti-*T. cruzi* activity at concentrations of 145.5 and 228 µg/mL, 57.5 and 99.5 µg/mL, and 102 and 467.5 µg/mL, respectively, after serial dilution in DMSO. The main chemical components of *S. aromaticum* L., *O. basilicum* L., and *A. millefolium* L. are eugenol, linalool, and chamazulene, respectively, which were attributed with the trypanocidal activity. Previous studies have shown the trypanocidal activity of *S. aromaticum* L. [26], *O. basilicum* L. [27], and *A. millefolium* L. [28] essential oils.

The essential oil of *Piper cubeba* L. [29] presented in vitro trypanocidal activity against *T. cruzi* at concentrations of 45.5 and 87.9 µg/mL, being serially diluted in DMSO and evaluated by the MTT colorimetric assay [15,16]. Analysis of the essential oil of this species revealed that sabinene, eucalyptol, 4-terpineol, β-pinene and camphor were the main components and were considered responsible for the observed trypanocidal activity. 

The essential oils of *Chenopodium ambrosioides* L., *Justicia pectoralis* Jacq., and *Vitex agnus-castus* L. [13] present in vitro anti-*T. cruzi* activity at concentrations of 21.3, 56.8, and 155.8 µg/mL, respectively. Each was dissolved in DMSO and further analyzed by colorimetric assay (MTT) [15,16]. The compound 1,8-cineole is the largest constituent of the *V. agnus-castus* essential oil, and terpinolene is a major component of the essential oils obtained from *J. pectorals* and *C. ambrosioides.*

The essential oils of the species *Lippia pedunculosa* Hayek [30], *Lippia sidoides* Cham. [13], *Lippia alba* (Mill.)*, Lippia citriodora* Kunth., *Lippia dulcis* Trev., *Lippia micromera* Schauer and *Lippia origanoides* Kunth. [31] showed in vitro trypanocidal activity against *T. cruzi*, with *L. pedunculosa* at concentrations of 11.3 and 15.1 µg/mL, *L. sidoides* at 10.3 and 28.9 µg/mL, *L. alba* at 5.5 and 12.5 µg/mL, *L. citriodora* at 24.3 and 75 µg/mL, *L. dulcis* at 32.8 and 51.7 µg/mL, and *L. micromera* at 50.6 and 60.7 µg/mL. Finally, *L. origanoides* obtained values of 9.9 and 50.5 µg/mL. Analysis of these essential oils revealed citral, *p*-cymene, carvacrol, limonene, carvone, thymol, *trans*-β-caryophyllene, rotundifolone and piperitenone as the principal constituents of these *Lippia* spp., which are rich sources of biologically active compounds.

Costa et al. (2013) [21] conducted studies with the essential oils of the species *Annona pickelii* (Diels) H. Rainer and *Annona salzmannii* A. DC., which presented in vitro anti-*T. cruzi* activity of 28.7 and 89.7 µg/mL, respectively. In 2012, Costa et al. [32] conducted a study on *Annona vepretorum* Mart., which presented trypanocidal activity against *T. cruzi* at a concentration of 40.9 µg/mL. Analysis of the essential oils of these species revealed that sesquiterpenes are the main constituents; for *Annona pickelii,* the highest percentages were for bicyclogermacrene and (*E*)-caryophyllene; for *A. salzmannii,* the compounds with the highest percentages were (*E*)-caryophyllene, bicyclogermacrene, δ-cadinene, α-copaene and germacrene D; and for *Annona vepretorum,* the compounds with the highest percentages were bicyclogermacrene and spathulenol. In general, the trypanocidal activities were attributed to the high concentrations of bicyclogermacrene.

Bay et al. [33] investigated the trypanocidal activity of the essential oils of four species of Annonaceae (*Bocageopsis multiflora*, *Duguetia quitarensis*, *Fusaea longifolia* and *Guatteria punctata*) against trypomastigote and intracellular amastigote forms of *T. cruzi*. The trypanocidal action results indicated that the essential oils were active, and that the essential oil of *G. punctata* was the most active (the main constituents for this oil were germacrene D, (*E*)-nerolidol and (*E*)-caryophyllene), with an IC_50_ = 0.029 μg/mL, which presented the highest selectivity index (SI) and was 34 times more effective than benznidazole (1 μg/mL). 

Study of Sainz et al. (2019) [34] with *Artemisia pedemontana* subsp. *assoana* (Willk.) Rivas Mart. (1,8-cineole and camphor as main constituents), experimentally cultivated in the greenhouse and aeroponically, against *T. cruzi* epimastigote forms showed moderate trypanocidal activity. 

Gutierrez et al. 2016 [35], examined the chemical analysis, antimicrobial activity and cytotoxic effects of OEs from *Piper aduncum* var. *ossanum* that was harvested from two locations, Bauta and Ceiba, Artemisa Province, Cuba. Both OEs showed the same activity against *T. cruzi* (approximately 8 μg/mL). 

KIAN et al. (2018) [36] revealed that kaurenoic acid was extracted from *Sphagneticola trilobata*. (L.) Pruski, and at the concentrations of 5, 10, 15, and 20 μg/mL, it showed strong trypanocidal activity against *T. cruzi*.

Recently, the inhibitory properties and cellular effects of the essential oils of *L. alba* and their main bioactive terpenes and the synergy between them were shown against the strains of *T. cruzi*. The *L. alba* OEs had significant differences in their chemical composition and trypanocidal performance (*p* = 0.0001). Citral chemotype oils showed greater trypanocidal activity than carvone essential oils, with 50% inhibitory concentrations (IC_50_ values) of 14 ± 1.5, 22 ± 1.4 and 74 ± 4.4 μg/mL in epimastigotes, trypomastigotes and amastigotes, respectively [37].

Pereira et al. (2018) [38] verified the leishmanicidal and anti-*T. cruzi* potential as well as the cytotoxicity of the *Alpinia speciosa* K. Schum. essential oil. *A. speciosa* presented 1,8-cineole (28.46%), camphor (17.10%) and sabinene (9.95%) as the main constituents. The cytotoxic activity of the essential oil showed a low value, while the antipromastigote and antiepimastigote activities showed values that were considered clinically relevant, with values below 500 μg/mL.

Zanusso Júnior et al. (2018) [39] investigated the activity of the EO of *Syzygium aromaticum* (main compounds are eugenol and β-caryophyllene) alone and in association with benznidazole (BZ) in mice infected with *T. cruzi* AM14 strain (TcIV) (considered resistant to BZ *in vivo*). When compared to untreated animals, experiments with the EO of *S. aromaticum* alone promoted a reduction of the parasitemia. However, the animals treated with BZ alone or in association showed a more significant reduction in parasitemia. 

In another study, Oliveira de Souza et al. (2017) [40] investigated the in vitro activity of OEs from leaves of *Eugenia brejoensis* Mazine (main compounds are δ-cadinene, *trans*-caryophyllene and α-muurolol), *Hyptis pectinata* (L.) Poit. (*trans*-caryophyllene, caryophyllene oxide and spathulenol), *Hypenia salzmannii* (Benth.) Harley (xanthoxylin, *trans*-caryophyllene and methyleugenol), *Lippia macrophylla* Cham. (thymol, carvacrol and σ-cymene) and seeds of *Syagrus coronata* (Mart.) Beccari (octanoic acid, dodecanoic acid and decanoic acid as major components) against *T. cruzi* epi- and trypomastigote and intracellular amastigote forms. The EO of *E. brejoensis* presented the best activity against *T. cruzi*, with selectivity indexes (SI) of 14.45 and 20.11 for trypomastigote and amastigote forms, respectively.

Estevam et al. (2017) [41] investigated the trypanocidal activity of the EO of *Protium ovatum* Engl. against *T. cruzi* trypomastigotes (IC_50_ = 28.55 μg/mL). The main compounds found in the EO were spathulenol, caryophylene oxide, β-caryophylene, and myrcene. However, the EO demonstrated moderate cytotoxicity against LLCMK_2_ adherent epithelial cells (CC_50_ = 150.9 μg/mL).

Tasdemir et al. (2019) [42] evaluated the in vitro activity of the essential oil of Turkish *Origanum onites* L. and its main constituents against *T. cruzi*. The main components found in the essential oil were carvacrol (70.6%), followed by linalool (9.7%), *p*-cymene (7%), γ-terpinene (2.1%), and thymol (1.8%). The EO and tested compounds have no inhibitory activity against *T. cruzi*.

Gutierrez et al. (2019) [43] studied the activity of the OE of *Phania matricarioides* (Spreng.) Griseb. against *T. cruzi* trypomastigotes. The main compounds identified in the EO were lavandulyl acetate and thymyl isobutyrate. The OE was bioactive against *T. cruzi* (IC_50_ = 2.2 µg/mL and SI = 13). Table 1 shows essential oils with trypanocidal activity against *T. cruzi.*

### 2.2. Essential Oils with Trypanocidal Activity against T. brucei.

Gutierrez et al. (2019) [43] studied the activity of the OE of *Phania matricarioides* (Spreng.) Griseb. against *T. brucei* trypomastigotes. The main compounds identified in the EO were lavandulyl acetate and thymyl isobutyrate. The inhibitory action of the EO against *T. brucei* was observed at an IC_50_ = 8.0 µg/mL (SI = 4.0).

In a more recent publication, Kpoviessi et al. (2014) [44] presented results that confirm the activity of citral found in the species *Cymbopogon citratus* (DC.) Stapf against *T. brucei*. Kpoviessi et al. (2014) also presented positive results for the in vitro antiparasitic activity of essential oils of the same genus: *Cymbopogon giganteus* Chiov, *Cymbopogon nardus* L., and *Cymbopogon schoenantus* L. Spreng. at concentrations of 0.25, 5.71 and 2.10 μg/mL, respectively, when dissolved in DMSO and analyzed using the colorimetric MTT assay [15,16].

The study presented by Nibret and Wink (2010) [46] was compared to that of Costa et al. (2013) [21] where the analysis of the essential oil of *Cinnamomum verum* J. Presl was made against the *T cruzi* species. (*E*)-cinnamaldehyde was effective against *T. brucei* trypomastigotes in low concentrations (2.93 μg/mL). The trypanocidal activities of the essential oils of *Hagenia abyssinica* (Bruce) J.F. Gmel. (Rosaceae), *Leonotis ocymifolia* (Burm. F.) Iwarsson var. (Lamiaceae) and *Moringa stenopetala* (Baker f.) Cufod. (Moringaceae), with concentrations of 42.30 mg/mL, 15.41 mg/mL and 5.03 mg/mL, respectively, were investigated. The analysis of the chemical composition of the oils identified ledol in *H. abyssinica*, caryophyllene oxide in *L. ocymifolia,* and benzyl isothiocyanate and isobutyl isothiocyanate in *M. stenopetala*. The cytotoxic and trypanocidal activities of these oils can be attributed to these components, and their biological activity can also be increased by the presence of other compounds acting in an additive or synergistic way.

The essential oil from the species *Keetia leucantha* (K. Krause) Bridson [47] presented in vitro anti-*T. brucei* activity at a concentration of 20.9 µg/mL. Analysis of the essential oil suggested α-ionone, β-ionone, and ursolic acid were the compounds that presented the best inhibitory activity against *T. brucei*, although analysis of the oil revealed that the principal constituents of the oil were *n*-hexadecanoic acid and phytol, which presented IC_50_ values of >100 and 19.1 µg/mL, respectively.

The *Ocimum gratissimum* L. essential oil [48] was active against *T. brucei* (in vitro) at a concentration of 1.66 µg/mL when dissolved in DMSO and evaluated using the MTT colorimetric assay [15,16]. Analysis of the essential oil of this species revealed that sabinene, eucalyptol, 4-terpineol, β-pinene and camphor are the main components and are considered responsible for the trypanocidal activity.

In another study, the essential oil of Aframomum sceptrum (Oliv. and D. Hanb.) K. Schum. [49] presented in vitro anti-*T. brucei* activity at a concentration of 1.51 µg/mL. Analysis of the essential oils of this species revealed the constituents β-pinene and caryophyllene oxide, to which the trypanocidal activity was attributed.

The *Kadsura longipedunculata* Finet el Gagnep (Nanwuweizi) essential oil [50] presents in vitro anti-*T. brucei* activity at the concentration of 50.52 µg/mL. This evaluation was made using the resazurin dye test [51] to evaluate trypanosome proliferation. Analysis of the essential oils revealed δ-cadinene and camphene as the principal components, which were attributed to the trypanocidal properties.

De Sousa et al. (2016) [52] investigated the EO of *Mentha crispa* L. and its main constituents (rotundifolone and four related *p*-menthane monoterpenes) against *T. brucei* trypomastigotes. The essential oil, the compounds rotundifolone and perillyl aldehyde presented a dose-dependent action and identical 50% growth inhibitory concentration (GI_50_) of 0.3 µg/mL.

Petrelli et al. (2016) [53] evaluated the biological activity exhibited by the essential oil obtained from the aerial parts of *Croton floribundus* Spreng against *T. brucei* in vitro. The proliferation of *T. brucei* was inhibited with IC_50_ values of 33.5 µg/mL from the essential oil and 5.6 µg/mL from the active component limonene. 

Gutierrez et al. 2016 [35], also examined the chemical analysis, antimicrobial activity and cytotoxic effects of OEs of *Piper aduncum* var. *ossanum* against *T brucei*, and showed an activity similar to *T cruzi* (approximately 8 μg/mL).

A study by Sobeh et al. (2016) [54] analyzed the composition of the *Eugenia uniflora* L. essential oil, to which *T. brucei* was highly susceptible, with an IC_50_ of 11.20 μg/mL and an SI of 6.82.

*Smyrnium olusatrum* L. (Apiaceae) is characterized by oxygenated sesquiterpenes containing a furan ring. In the work of Petrelli et al. 2017 [55], the *T. brucei* inhibitory activities of the essential oils obtained from different organs and of the main oxygenated sesquiterpenes, such as isofuranediene, germacrone and β-acetoxifuranoeudesm-4-ene, were explored. All oils inhibited the growth of the parasite, showing IC_50_ values of 1.9–4.0 µg/mL; isofuranediene exhibited significant and selective inhibitory activity against *T. brucei* (IC_50_ of 0.6 μg/mL, SI = 30), with β-acetoxifuranoeudesm-4-ene giving a moderate potentiating effect.

Costa et al. (2018) [56] tested 17 oils, and three showed high anti-*T. brucei* activity (IC_50_ values <10 μg/mL): *Juniperus oxycedrus* L. (IC_50_ of 0.9 μg/mL), *Cymbopogon citratus* L. (IC_50_ of 3.2 μg/mL) and *Lavandula luisieri* L. (IC_50_ of 5.7 μg/mL). These oils did not have cytotoxic effects on macrophages, presenting the high of the selectivity index values (63.4, 9.0 and 11.8, respectively).

Evaluation of the inhibitory effects of Apiaceae essential oils against *T. brucei* in the study by Ngahang Kamte et al. (2018) [57] showed that the oils of some species (*Echinophora spinosa* L., *Sison amomum* L., *Crithmum maritimum* L. and *Helosciadium nodiflorum* (L.) Koch) were active, with EC_50_ values in the range of 2.7–10.7 μg/mL.

Kamte et al. (2017) [58] evaluated the trypanocidal activity of EO of six medicinal and aromatic plants (*Azadirachta indica*, *Aframomum melegueta*, *Aframomum daniellii*, *Clausena anisata*, *Dichrostachys cinerea* and *Echinops giganteus*) against *T. brucei* trypanosomes. The EOs of *A. indica*, *A. daniellii* and *E. giganteus* presented IC_50_ values of 15.21, 7.65 and 10.50 µg/mL, respectively, while *A. melegueta*, *C. anisata* and *D. cinerea* did not show activity. Sesquiterpene hydrocarbons, monoterpene hydrocarbons, and oxygenated sesquiterpenes were the main compounds found in the EOs.

Hoet et al. (2006) [59] analyzed the essential oil from the leaves of *Strychnos spinosa* (Loganiaceae). Twenty-two compounds were identified in the oil; the main constituents were palmitic acid (34.3%) and linalool (16.0%). The in vitro activity of the essential oil and 15 components against *T. brucei brucei* and mammalian cells were analyzed. The essential oil was active against the parasite without a high selectivity [IC_50_ in *T. b. brucei* = 13.5 µg/mL, SI = 4.4]. (*E*)-Nerolidol and linalool showed a more potent and selective effect on trypanosomes [IC_50_ = 1.7 and 2.5 µg/mL (7.6 and 16.3 µM).

Tasdemir et al. (2019) [42] also evaluated the in vitro activity of the essential oil of *Origanum onites* L. turco and its main constituents against *T. brucei rhodesiense*. The oil showed significant in vitro activity against *T. brucei rhodesiense* (IC_50_ 180 ng/mL) without causing toxicity in mammalian cells. In the in vivo model against *T. brucei brucei*, thymol extended the animals survival.

Binh Le et al. (2019) [60] analyzed thirty-seven Vietnamese essential oils (OE) against *T. brucei brucei* (Tbb) and cytotoxicity in mammalian cells (WI38, J774). The results showed a selective effect of four OEs extracted from three species of Zingiberaceae (*Curcuma longa, Curcuma zedoaria* and *Zingiber officinale*) and one species of Lauraceae (*Litsea cubeba*) with an IC_50_ of 3.17 ± 0.72, 2.51 ± 1.08, 3.10 ± 0.08 and 2.67 ± 1.12 nL/mL, respectively, and SI > 10. Table 2 shows essential oils with trypanocidal activity against *T. brucei.*

### 2.3. Essential Oils with Trypanocidal Activity against T. evansi.

According to Carmo et al. (2015) [61] and Baldissera et al. (2014) [62], the essential oil from the species *Achyrocline satureioides* (Lam.) DC. (Macela) presented bioactivity in vivo against *T. evansi* at a dose of 1.5 mL/kg after oral administration for five consecutive days. For hematological analysis, blood samples were collected on the fifth day. The chemical compounds with higher proportions in the essential oil were the terpenes α-pinene and β-caryophyllene, and the trypanocidal activity was attributed to these compounds.

The essential oil of *Aniba canelilla* (HBK) Mez, popularly known as “bark-precious” (precious bark), presented as a major constituent 1-nitro-2-phenylethane, a rare molecule in plants, and methyleugenol, and the oil presented results against *T. evansi*. Methyleugenol was slightly more active than 1-nitro-2-phenylethane, and in vitro studies showed that the oil extracted from the stems of *A. canelilla* can be considered a potential natural treatment for trypanosomiasis [63].

Baldissera et al. (2013) [64] investigated the in vitro activity of the essential oils of andiroba (*Carapa guaianensis*) and aroeira (*Schinus molle*) against *T. evansi* trypanosomes using conventional (at concentrations of 0.5%, 1.0%, and 2.0%) and nanoemulsion forms (at concentrations of 0.5% and 1.0%). The tests were performed in duplicate and the numbers of parasites evaluated after 1, 3 and 6 h. The trypanocidal activity of essential oils was dose-dependent and, after 6 h of experiment, no living trypanosomes were observed. For the nanoemulsion oils, the activity was dose-dependent after 1 h of interaction, but after 3 h no alive parasites were observed.

Baldissera et al. (2017) [65] verified the trypanocidal activity in vitro of EOs of *Lippia alba* and *Lippia origanoides* against *T. evansi* trypomastigotes. The lower concentration of EOs (0.5%) eliminated the parasites after 6 h of interaction. Experiments in vivo with mice also were performed. The animals were treated with EOs at a dose of 1.5 mL/kg for five days and, despite not showing curative efficacy, the treated mice with EO of *L. origanoides* showed increase in longevity when compared to the control group. Table 3 shows essential oils with trypanocidal activity against *T. evansi.*

## 3. Proposed Mechanisms of Action for the Trypanocidal Activity of Essential Oils

Essential oils from aromatic plants and their major components have been studied for their antimicrobial activities, with significant anthelmintic and antiprotozoal activity [66]; nevertheless, their mechanism of action remains poorly studied. Therefore, identification of the active components of EOs and understanding their mechanisms of action are essential.

**Figure 2:**
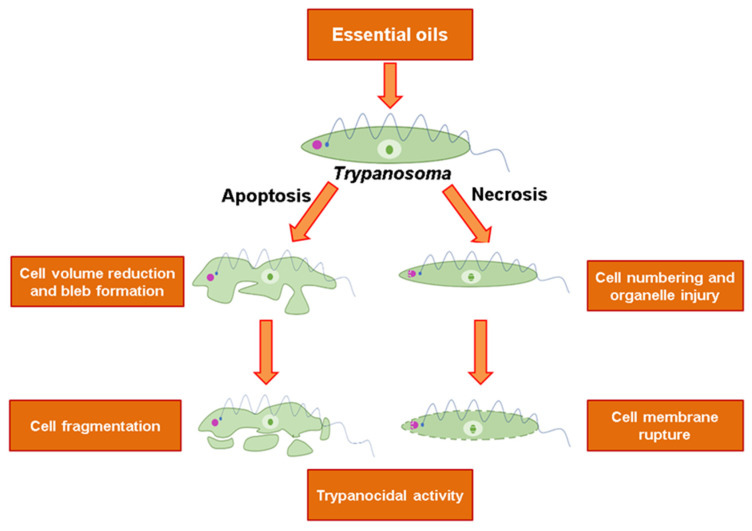
Main mechanisms of the trypanocidal action of essential oils. Source: Adapted from Grivicich, I.; Regner, A.; d. Rocha, A.B., 2007 [72].

The activity of some EOs could be associated with the lipophilic characteristics of their constituents. Lipophilic molecules can cross the cell membrane, and once inside the cells, these molecules can interact with a variety of proteins, inactivating enzymes and affecting cellular activities [67]. Depolarization of the mitochondrial membrane is associated with changes in calcium channels and ROS generation that can trigger cell death by apoptosis and necrosis [68,69] (Figure 2).

A discontinuous plasma membrane, indicative of a loss of integrity of the parasite, is a major feature of cell death by necrosis. In this type of cell death, there are also alterations to the mitochondria, ATP depletion, generation of reactive oxygen species, and cytoplasm vacuolization [70]. The essential oils of *Melaleuca alternifolia* [61], *Xylopia frutescens* [20], *Xylopia laevigata* [20], *Cymbopogon citratus* [44,61], *Cymbopogon giganteus* [44], *Cymbopogon nardus* [44], and *Cymbopogon schoenantus* [44] presented this type of action.

During apoptosis, there are alterations such as cytoplasmic blebbing, cell volume reduction, loss of mitochondrial membrane potential, condensation of nuclear chromatin, and DNA fragmentation [71]. Such characteristics were observed from the essential oils of *Cinnamodendron dinisii* [14], *Siparuna guianensis* [14], *Cinnamomum verum* [19], *Lippia dulcis* [30], *Achyrocline satureioides* [61,62], *Lippia sidoides* [13], *Lippia origanoides* [13,30,65], *Chenopodium ambrosioides* [13], *Justicia pectorales* [13], *Lippia citriodora* [30], *Lippia pedunculosa* [31] and *Lippia alba* [30]. However, due to the large number of components and potential synergistic and/or antagonistic interactions between them, it is likely that in addition to membranes, essential oils can also act against other cellular targets (Figure 2).

Despite the various pharmacological properties attributed to essential oils, and prospects for clinical application, especially in the area of psychopharmacology due to the relaxing effects of essential oils traditionally used for therapeutic purposes, there are few reports of toxicological studies of essential oils. Therefore, it is not possible to describe the possible side effects. Thus, it is important to advance in the knowledge about the therapeutic safety of these natural products [72].

## 4. Methodology

The present study was based on works published on essential oils with trypanocidal activity in experimental models. The search was performed on the PubMed Scientific Database (Home-PubMed-NCBI) in November 2019. For the selection of data in English, search terms related to the theme were used, such as “essential oils” and “*Trypanosoma*” or “trypanocidal”. Articles that are literature reviews or contain only oil constituents were not included in the review.

## 5. Conclusions

The data presented demonstrate the importance of essential oils as natural products to be investigated for the development of new therapeutic options in the face of neglected diseases, especially trypanosomiasis. It is necessary to evaluate the main chemical components of these oils in experimental models in vitro and in vivo, in addition to researching their mechanism of trypanocidal action. In addition, the therapeutic safety of these products must be evaluated through toxicological studies. The abundance of essential oils in several tropical countries and low production costs make them interesting compounds to be researched as new pharmacological tools for use in the treatment of neglected diseases. The study will also support the discovery of new essential oils and/or chemical constituents, as well as analogous compounds with trypanocidal activity.

## Figures and Tables

**Table 1:** Essential oils with trypanocidal activity against *T. cruzi.*

Plant	Experimental Models	Dose/ Concentration	Major Constituent(s)
***Achillea millefolium* L.** [25]	In vitro (Microdilutions/Culture)	145.5 µg/mL 288 µg/mL	chamazulene
***Aloysia triphylla* (L’Hér.) Britton** [23]	In vivo (Orogastric tube)	100 mg/kg 250 mg/kg	Citral
***Alpinia speciosa* K. Schum** [38]	In vitro (Microdilutions/Culture)	67.18 μg/mL	1,8-cineole camphor sabinene
***Annona salzmannii* A. DC** [21]	In vitro (Microdilutions/Culture)	89.7 µg/mL	δ-cadinene *E*-caryophyllene α-copaene bicyclogermacrene germacrene D
***Annona pickelii* (Diels) H. Rainer** [21]	In vitro (Microdilutions/Culture)	27.2 µg/mL	bicyclogermacrene *E*-caryophyllene α-copaene α-humulene
***Annona vepretorum* Mart** [32]	In vitro (Microdilutions/Culture)	31.9 µg/mL	bicyclogermacrene spathulenol α-phellandrene α-pinene (*E*)-β-ocimene germacrene D *p*-cymene
***Artemisia absinthium*** [16]	In vitro (Microdilutions/Culture)	1 µg/mL 10 µg/mL 100 µg/mL	*cis*-epoxyocimene (–)-*cis*-chrysanthenol dihydrochamazulene
***Artemisia pedemontana* subsp.*****assoana*****(Willk.) Rivas Mart** [34]	In vitro (Microdilutions/Culture)	100 µg/mL	1,8-cineole
***Bocageopsis multiflora* Mart** [33]	In vitro (Microdilutions/Culture)	0.46 μg/mL	linalool, 1-epi-cubenol β-bisabolene spathulenol
***Chenopodium ambrosioides* L.** [13]	In vitro (Microdilutions/Culture)	21.3 μg/mL 28.1 μg/mL	terpinolene
***Cinnamodendron dinisii* Schwacke** [15]	In vitro (Microdilutions/Culture)	209.3 µg/mL 282.9 µg/mL	monoterpene hydrocarbons, sesquiterpene, hydrocarbons, α-pinene, β-pinene, sabinene, bicyclogermacrene.
***Cinnamomum verum* J. Presl** [19]	In vitro (Microdilutions/Culture)	5.05 μg/mL 20 µg/mL 24.13 µg/mL	(*E*)-cinnamaldehyde eugenol
***Cymbopogon citratus* (DC.) Stapf** [17,44]	In vitro (Microdilutions/ Culture)	15.5 µg/mL 126.5 µg/mL 3.2 μg/mL	citral
***Dracocephalum kotschyi*** [45]	In vitro (Microdilutions/Culture)	6.2 μM	geranial limonene 1,1-dimethoxy-decane
***Duguetia quitarensis* Benth** [44]	In vitro (Microdilutions/Culture)	0.46 μg/mL	4-heptanol α-thujene (*E*)-caryophyllene
***Eugenia*** ***brejoensis* Mazine** [40]	In vitro (Microdilutions/Culture)	29 ± 4.5 µg/mL (Epimastigote) 17.39 ± 0.62 (Trypomastigote) 12.5 ± 1.74 (Amastigote)	δ-cadinene *trans*-caryophyllene α-muurolol
***Fusaea longifolia* (Aubl.) Saff** [33]	In vitro (Microdilutions/Culture)	0.3 μg/mL	β-selinene *cis*-β-guaiene (*Z*)-α-bisabolene (*E*)-caryophyllene
***Guatteria punctata* (Aubl.) RA Howard** [33]	In vitro (Microdilutions/Culture)	0.029 μg/mL	germacrene D (*E*)-nerolidol (*E*)-caryophyllene
***Hyptis pectinata* (L.) Poit** [40]	In vitro (Microdilutions/Culture)	56.1 ± 17 µg/mL (Epimastigote) 25.64 ± 3.26 µg/mL (Trypomastigote) 25.5 ± 0.5 µg/mL (Amastigote)	*trans*-caryophyllene caryophyllene oxide spathulenol
***Hypenia salzmannii* (Benth.) Harley** [40]	In vitro (Microdilutions/Culture)	42.13 ± 9.34 µg/mL (Epimastigote) 36.27 ± 3.2 µg/mL (Trypomastigote) 35.25 ± 3.07 µg/mL (Amastigote)	xanthoxylin *trans*-caryophyllene methyl-eugenol
***Justicia pectorales* Jacq** [13]	In vitro (Microdilutions/Culture)	44.5 µg/mL 56.8 µg/mL	terpinolene
***Lantana camara* L.** [18]	In vitro (Microdilutions/Culture)	201.94 μg/mL	(*E*)-caryophyllene bicyclogermacrene germacrene D terpinolene sabinene
***Lippia macrophylla*** **Cham.** [40]	In vitro (Microdilutions/Culture)	37.6 ± 5 µg/mL (Epimastigote) 70.6 ± 7.5 µg/mL(Trypomastigote) 51.76 ± 5.65 µg/mL (Amastigote)	thymol carvacrol cymene
***Lippia sidoides* Cham.** [13]	In vitro (Microdilutions/Culture)	10.3 µg/mL 28.9 µg/mL	thymol
***Lippia pedunculosa* Hayek.** [31]	In vitro (Microdilutions/Culture)	11.3 µg/mL 15.1 µg/mL	(*R*)-limonene rotundifolone piperitenone
***Lippiaalba* (Mill.)** [30,37]	In vitro (Microdilutions/Culture)	5.5 µg/mL 12.2 µg/mL 14.0 μg/mL 22 μg/mL 74 μg/mL	citral *trans-*β-caryophyllene limonene carvone
***Lippia*** ***citriodora* Kunth.** [30]	In vitro (Microdilutions/Culture)	24.3 µg/mL 75 µg/mL	citral *trans-*β-caryophyllene
***Lippia*** ***dulcis* Trev.** [30]	In vitro (Microdilutions/Culture)	32.8 µg/mL 51.7 µg/mL	citral *trans-*β-caryophyllene
***Lippia micromera* Schauer** [30]	In vitro (Microdilutions/Culture)	50.6 µg/mL 60.7 µg/mL	*p*-cymene carvacrol thymol
***Lippia origanoides* Kunth.** [13,30]	In vitro (Microdilutions/Culture) In vivo (Intramuscular injection)	9.9 µg/mL 50.5 µg/mL 0.5, 1.0 and 2.0% 1.5 mL kg^−1^	carvacrol thymol *p*-cymene
***Ocimum basilicum* L.** [25]	In vitro (Microdilutions/Culture)	102 µg/mL 467.5 g/mL	linalool
***Origanum vulgare* L.** [22]	In vitro (Microdilutions/Culture)	115 µg/mL 175 µg/mL	3-cycle-hexen-1-ol
***Origanum onites* L.** [42]	In vitro (Microdilutions/Culture)	>90 μg/mL	carvacrol linalool *p*-cymene γ-terpinene thymol
***Phania matricarioides* (Spreng.) Griseb.** [43]	In vitro (Microdilutions/Culture)	2.2 µg/mL	lavandulyl acetate thymyl isobutyrate
***Piper aduncum* var.** ***ossanum*** [35]	In vitro (Microdilutions/Culture)	8.0 µg/mL 8.6 µg/mL	camphene camphor piperitone viridiflorol
***Piper cubeba* L.** [29]	In vitro (Microdilutions/Culture)	45.5 µg/mL 87.9 µg/mL	sabinene, eucalyptol 4-terpineol pinene camphor
**Protium ovatum Engl.** [41]	In vitro (Microdilutions/Culture)	28.55 μg/mL	spathulenol caryophyllene oxide β-caryophyllene myrcene
***Siparuna guianensis* Aublet** [15]	In vitro (Microdilutions/Culture)	209.3 µg/mL 282.9 µg/mL	β-myrcene germacrene-D bicyclogermacrene
***Sphagneticola trilobata* (L.) Pruski.** [36]	In vitro (Microdilutions/Culture)	5, 10, 15, and 20 μg/mL	kaurenoic acid
***Syzygium aromaticum* L.** [25], [39]	In vitro (Microdilutions/Culture) In vivo (Administered orally)	57.5 µg/mL 99.5 µg/mL 100 mg/kg/day	eugenol
***Syagrus coronata* (Mart.) Beccari** [40]	In vitro (Microdilutions/Culture)	100.6 ± 38 µg/mL (Epimastigote) 182.49 ± 58.05µg/mL (Trypomastigote) 408.33 ± 23.36 µg/mL (Amastigote)	octanoic acid, dodecanoic acid decanoic acid.
***Thymus*** ***vulgaris* L.** [25]	In vitro (Microdilutions/Culture)	38 µg/mL 77 µg/mL	Thymol
***Xylopia laevigata* (Mart.)** [20]	In vitro (Microdilutions/Culture)	12.7 μg/mL 22.2 μg/mL 13.4 μg/mL 27.7 μg/mL	Germacrene bicyclogermacrene β-elemene δ-selinene (*E*)-caryophyllene germacrene γ-muurolene
***Xylopia frutescens* Aubl.** [20]	In vitro (Microdilutions/Culture)	11.9 μg/mL 20.2 μg/mL	(*E*)-caryophyllene bicyclogermacrene germacrene β-elemene (*E*)-β-ocimene
***Vitex agnus-castus* L.** [13]	In vitro (Microdilutions/Culture)	155.8 µg/mL	terpinyl acetate 1,8-cineole

**Table 2:** Essential oils with trypanocidal activity against *T. brucei.*

Plant	Experimental Models	Dose/ Concentration	Major Constituent(s)
***Aframomum sceptrum* (Oliv. and D.Hanb.) K. Schum.** [49]	In vitro (Microdilutions/Culture)	1.5 µL/mL	β-pinene caryophyllene oxide cyperene
***Aframomum daniellii* (Hook. F.) K. Schum.** [58]	In vitro (Microtiter/Culture)	7.65 µg/mL	sabinene (*E*)-caryophyllene
***Azadirachtaindica* A. Juss.** [58]	In vitro (Microtiter/Culture)	15.21 ± 0.97 µg/mL	germacrene B γ-elemene β-elemene (*E*)-caryophyllene
***Crithmum maritimum* L.** [57]	In vitro (Microdilutions/Culture)	5.0 ± 0.8 µg/mL	limonene γ-terpinene sabinene
***Croton floribundus* Spreng.** [53]	In vitro (Microdilutions/Culture)	33.5 µg/mL with the essential oil, 5.6 µg/mL with the limonene	spathulenol caryophyllene oxide limonene.
***Curcuma*** ***longa*****L.** [60]	In vitro (Microdilutions/Culture)	3.17 ± 0.72 ng/mL	α-zingiberene β-bisabolene β-sesquiphellandrene *ar*-curcumene
***Curcuma*** ***zedoaria*** [60]	In vitro (Microdilutions/Culture)	2.51 ± 1.08 ng/mL	8,9-Dehydro-9-formyl cycloisolongifolene curdione germacrone
***Cymbopogon citratus* (DC.) Stapf** [56]	In vitro (Microdilutions/Culture)	3.2 μg/mL	citral
***Cymbopogon giganteus* Chiov** [44]	In vitro (Microdilutions/Culture)	0.25 µg/ml	*trans-p*-mentha-1(7),-dien-2-ol *trans*-carveol, *trans-p*-mentha-2,8-dienol *cis*-*p*-mentha-2,8-dienol *cis*-*p*-mentha-1(7),8-dien-2-ol limonene *cis*-carveol *cis*-carvone
***Cymbopogon*** ***nardus* L.** [44]	In vitro (Microdilutions/Culture)	5.71 µg/mL	β-citronellal nerol β-citronellol elemol limonene
***Cymbopogon schoenantus* L. Spreng.** [44]	In vitro (Microdilutions/Culture)	2.10 µg/mL	piperitone ( + )-2-carene limonene elemol β-eudesmol
***Echinophora spinosa* L.** [56]	In vitro (Microdilutions/Culture)	2.7 ± 0.6 μg/mL	myristicin terpinolene (*Z*)-falcarinol
***Echinophora spinosa* L.** [56]	In vitro (Microdilutions/Culture)	4.0 ± 1.6 μg/mL	α-phellandrene *p*-cymene β-phellandrene *E,E*-2,6-dimethyl-1,3,5,7-octatetraene α-pinene
***Echinops giganteus* var. *lelyin* C. D. Adams** [58]	In vitro (Microdilutions/Culture)	10.50 µg/mL	silphiperfol-6-ene presilphiperfolan-8-ol cameroonan-7-α-ol
***Erigeron floribundus* (Kunth) Schultz-Bip.** [53]	In vitro (Microdilutions/Culture)	33.5 µg/mL 5.6 µg/mL	spathulenol Caryophyllene oxide limonene
***Eugenia uniflora*. L.** [54]	In vitro (Microdilutions/Culture)	11.20 μg/mL	spathulenol
***Hagenia abyssinica* (Bruce) J.F. Gmel.** [46]	In vitro (Microdilutions/Culture)	42.30 µg/mL	ledol
***Helosciadium nodiflorum* (L.) Koch** [57]	In vitro (Microdilutions/Culture)	10.7 ± 4 µg/mL	myristicin (*z*)-β-ocimene
***Juniperus oxycedrus* L.** [56]	In vitro (Microdilutions/Culture)	0.9 μg/mL	monoterpene hydrocarbons
***Kadsura longipedunculata* Finet el Gagnep (Nanwuweizi)** [50]	In vitro (Microdilutions/Culture)	50.52 µg/mL	δ-cadinene, camphene borneol cubenol δ-cadinol
***Keetia*** ***leucanta* (K. Krause) Bridson** [47]	In vitro (Microdilutions/Culture)	20.9 µg/mL	*n*-hexadecanoic acid, phytol
***Lavandula luisieri* L.** [56]	In vitro (Microdilutions/Culture)	5.7 μg/mL	oxygen-containing monoterpenes
***Leonotis ocymifolia* (Burm. F.) Iwarsson var. *raineriana*** [46]	In vitro (Microdilutions/Culture)	15.41 µg/mL	caryophyllene oxide
***Litsea cubeba*****Pers** [60]	In vitro (Microdilutions/Culture)	2.67 ± 1.12 ng/mL	Citronellal isopulegol limonene pulegol linalool citronellol
***Mentha crispa* L.** [52]	In vitro (Microdilutions/Culture)	0.3 µg/mL 1 µg/mL	rotundifolone *p*-menthane monoterpenes (two stereoisomers of limonene epoxide, perillyl alcohol and perillyl aldehyde)
***Moringa stenopetala* (Baker f.) Cufod.** [46]	In vitro (Microdilutions/Culture)	5.03 µg/mL	benzyl isothiocyanate isobutyl isothiocyanate
***Ocimum gratissimum* L.** [13,48]	In vitro (Microdilutions/Culture)	1.66 µg/mL	*p*-cymene thymol γ-terpinene β-myrcene α-thujene
***Origanum onites* L.** [42]	In vitro (Microdilutions/Culture)	>90 μg / mL	carvacrol linalool *p*-cymene γ-terpinene thymol
***Phania matricarioides* (Spreng.) Griseb.** [43]	In vitro (Microdilutions/Culture)	8.0 µg/mL	lavandulyl acetate thymyl isobutyrate
***Piper aduncum* var. *ossanum*** [35]	In vitro (Microdilutions/Culture)	8.1 µg/mL 8.4 µg/mL	camphene camphor piperitone viridiflorol piperitone
***Sison amomum* L.** [57]	In vitro (Microdilutions/Culture)	4.3 ± 0.7 µg/mL	sabinene β-phellandrene germacrene D terpinen-4-ol γ-terpinene myrcene
***Smyrnium olusatrum* L.** [55]	In vitro (Microdilutions/Culture)	1.9–4.0 µg/mL 0.6 µg/mL	isofuranodiene germacrone β-acetoxyfuranoeudesm-4(15)-ene
***Strychnos spinosa* Lam.** [59]	In vitro (Microdilutions/Culture)	13.5 μg/mL	palmitic acid linalool (*E*)-nerolidol
***Zingiber officinale*****R.** [60]	In vitro (Microdilutions/Culture)	3.10 ± 0.08 ng/mL	α-zingiberene β-bisabolene β-sesquiphellandrene *ar*-curcumene

**Table 3:** Essential oils with trypanocidal activity against *T. evansi.*

Plant	Experimental Models	Dose/ Concentration	Major Constituent(s)
***Achyrocline satureoides* (Lam.) DC. (Macela)** [61,62]	In vivo (Administered orally)	1.5 mL/kg	α-pinene β-caryophyllene β- ocimene 1,8-cineole γ-eudesmol
***Aniba canelilla* (Kunth) Mez** [63]	In vitro (Microtiter/Culture)	0.5, 1.0 and 2.0% of the tested oil concentration	1-nitro-2-phenylethane Methyleugenol
***Carapa guaianensis* Aubl.** [61]	In vitro (Microdilutions/Culture)	0.5% 1.0%	
***Lippia alba* (Mill.) N.E. Br. ex Britton and P. Wilson** [62,65]	In vivo (Intramuscular injection)	0.5, 1.0 and 2.0% 1.5 mL/kg	citral *trans-*β-caryophyllene limonene carvone
***Lippia origanoides* Kunth** [62,65]	In vivo (Intramuscular injection)	0.5, 1.0 and 2.0% 1.5 mL/Kg	carvacrol thymol *p*-cymene
***Melaleuca alternifolia* Cheel** [61]	In vivo (Administered orally)	1 mL/kg	terpinen-4-ol γ-terpinene
***Schinus mole* L.** [64]	In vitro (Microdilutions/Culture)	0.5% 1.0%	mono and sesquiterpenes nonoxygenated

## References

[1] Silva R.A.M.S., Seidl A., Ramirez L., Dávila A.M.R. (2002). Tripanossoma Evansi e Tripanossoma Viváx: Biologia, Diagnóstico e Controle.

[2] Hotez P.J., Alvarado M., Basáñez M.G., Bolliger I., Bourne R., Boussinesq M., Brooker S.J., Brown A.S., Buckle G., Budke C.M. (2014). The Global Burden of Disease Study 2010: Interpretation and Implications for the Neglected Tropical Diseases. PLoS Negl. Trop. Dis..

[3] (2020). Trypanosomiasis. Britannica. https://www.britannica.com/science/brucellosis.

[4] Bottieau E., Clerinx J. (2019). Human African Trypanosomiasis Progress and Stagnation. Infect. Dis. Clin. NA..

[5] Simarro P.P., Cecchi G., Paone M., Franco J.R., Diarra A., Ruiz J.A., Fèvre E.M., Courtin F., Mattioli R.C., Jannin J.G. (2010). The Atlas of Human African Trypanosomiasis: A Contribution to Global Mapping of Neglected Tropical Diseases. Int. J. Health Geogr..

[6] Aregawi W.G., Agga G.E., Abdi R.D., Büscher P. (2019). Systematic Review and Meta-Analysis on the Global Distribution, Host Range, and Prevalence of Trypanosoma Evansi. Parasite. Vector..

[7] Joshi P.P., Shegokar V.R., Powar R.M., Herder S., Katti R., Salkar H.R., Dani V.S., Bhargava A., Jannin J., Truc P. (2005). Human Trypanosomiasis Caused by Trypanosoma Evansi in India: The First Case Report. Am. J. Trop. Med. Hyg..

[8] de Cássia da Silveira e Sá R., Lima T.C., da Nóbrega F.R., de Brito A.E.M., de Sousa D.P. (2017). Analgesic-like Activity of Essential Oil Constituents: An Update. Int. J. Mol. Sci..

[9] Sarmento-Neto J.F., Do Nascimento L.G., Felipe C.F.B., De Sousa D.P. (2016). Analgesic Potential of Essential Oils. Molecules.

[10] De Cássia Da Silveira E., Sá R., Andrade L.N., Pergentino De Sousa D. (2013). Molecules A Review on Anti-Inflammatory Activity of Monoterpenes. Molecules.

[11] Sobral M.V., Xavier A.L., Lima T.C., De Sousa D.P. (2014). Antitumor Activity of Monoterpenes Found in Essential Oils. Sci. World, J..

[12] De Assis Oliveira F., Andrade L.N., De Sousa É.B.V., De Sousa D.P. (2014). Anti-Ulcer Activity of Essential Oil Constituents. Molecules.

[13] Borges A.R., Aires J.R.d.A., Higino T.M.M., Medeiros M.d.G.F.d., Citó A.M.d.G.L., Lopes J.A.D., Figueiredo R.C.B.Q.d. (2012). Trypanocidal and Cytotoxic Activities of Essential Oils from Medicinal Plants of Northeast of Brazil. Exp. Parasitol..

[14] Andrade M.A., Cardoso M.D.G., Gomes M.d.S., de Azeredo C.M.O., Batista L.R., Soares M.J., Rodrigues L.M.A., Figueiredo A.C.S. (2015). Biological Activity of the Essential Oils from Cinnamodendron Dinisii and Siparuna Guianensis. Brazilian, J. Microbiol..

[15] Berridge M., Herst P., Tan A. (2005). Tetrazolium Dyes as Tools in Cell Biology: New Insights into Their Cellular Reduction. Biotechnol Annu Rev..

[16] González-Coloma A., Reina M., Sáenz C., Lacret R., Ruiz-Mesia L., Arán V.J., Sanz J., Martínez-Díaz R.A. (2012). Antileishmanial, Antitrypanosomal, and Cytotoxic Screening of Ethnopharmacologically Selected Peruvian Plants. Parasitol. Res..

[17] Santoro G., Cardoso M., Guimarães L., Freire J., Soares M. (2007). Anti-Proliferative Effect of the Essential Oil of Cymbopogon Citratus (DC) Stapf (Lemongrass) on Intracellular Amastigotes, Bloodstream Trypomastigotes and Culture Epimastigotes of Trypanosoma Cruzi (Protozoa: Kinetoplastida). Parasitology.

[18] Barros L.M., Duarte A.E., Morais-Braga M.F.B., Waczuk E.P., Vega C., Leite N.F., De Menezes I.R.A., Coutinho H.D.M., Rocha J.B.T., Kamdem J.P. (2016). Chemical Characterization and Trypanocidal, Leishmanicidal and Cytotoxicity Potential of Lantana Camara, L. (Verbenaceae) Essential Oil. Molecules.

[19] Azeredo C.M.O., Santos T.G., Maia B.H.L.d.N.S., Soares M.J. (2014). In Vitro Biological Evaluation of Eight Different Essential Oils against Trypanosoma Cruzi, with Emphasis on Cinnamomum Verum Essential Oil. BMC Complement. Altern. Med..

[20] Da Silva T.B., Menezes L.R.A., Sampaio M.F.C., Meira C.S., Guimarães E.T., Soares M.B.P., Do Prata A.P.N., De Nogueira P.C.L., Costa E.V. (2013). Chemical Composition and Anti-Trypanosoma Cruzi Activity of Essential Oils Obtained from Leaves of Xylopia Frutescens and X. Laevigata (Annonaceae). Nat. Prod. Commun..

[21] Costa E.V., Dutra L.M., Salvador M.J., Ribeiro L.H.G., Gadelha F.R., De Carvalho J.E. (2013). Chemical Composition of the Essential Oils of Annona Pickelii and Annona Salzmannii (Annonaceae), and Their Antitumour and Trypanocidal Activities. Nat. Prod. Res..

[22] Santoro G.F., Das Graças Cardoso M., Guimarães L.G.L., Salgado A.P.S.P., Menna-Barreto R.F.S., Soares M.J. (2007). Effect of Oregano (Origanum Vulgare, L.) and Thyme (Thymus Vulgaris, L.) Essential Oils on Trypanosoma Cruzi (Protozoa: Kinetoplastida) Growth and Ultrastructure. Parasitol. Res..

[23] Rojas J., Palacios O., Ronceros S. (2012). Efecto Del Aceite Esencial de Aloysia Triphylla Britton (Cedrón) Sobre El Trypanosoma Cruzi En Ratones. Rev. Peru. Med. Exp. Salud Publica.

[24] Stashenko E., Martínez J., Durán G.D., Díaz O. (2007). Estudio Comparativo de La Composición Química de Los Aceites Esenciales de Aloysia Triphylla L’her Britton Cultivada En Diferentes Regiones de Colombia. Sci. Tech..

[25] Santoro G.F., Cardoso M.G., Guimarães L.G.L., Mendonça L.Z., Soares M.J. (2007). Trypanosoma Cruzi: Activity of Essential Oils from Achillea Millefolium, L., Syzygium Aromaticum, L. and Ocimum Basilicum, L. on Epimastigotes and Trypomastigotes. Exp. Parasitol..

[26] Zheng G.Q., Kenney P.M., Lam L.K.T. (1992). Sesquiterpenes from Clove (Eugenia Caryophyllata) as Potential Anticarcinogenic Agents. J. Nat. Prod..

[27] Suppakul P., Miltz J., Sonneveld K., Bigger S. (2003). Antimicrobial Properties of Basil and Its Possible Application in Food Packaging. J. Agricul Food Chem.

[28] Rohloff J., Skagen E.B., Steen A.H., Iversen T.H. (2000). Production of Yarrow (Achillea Millefolium, L.) in Norway: Essential Oil Contente and Quality. J. Agric. Food Chem.

[29] Esperandim V.R., Ferreira D.d.S., Rezende K.C.S., Magalhães L.G., Souza J.M., Pauletti P.M., Januário A.H., Laurentz R.d.S.d., Bastos J.K., Símaro G.V. (2013). In Vitro Antiparasitic Activity and Chemical Composition of the Essential Oil Obtained from the Fruits of Piper Cubeba. Planta Med..

[30] Escobar P., Leal S.M., Herrera L.V., Martinez J.R., Stashenko E. (2010). Chemical Composition and Antiprotozoal Activities of Colombian Lippia Spp Essential Oils and Their Major Components. Mem. Inst. Oswaldo Cruz.

[31] Menezes L.R.A., Santos N.N., Meira C.S., Ferreira Dos Santos J.A., Guimarães E.T., Soares M.B.P., Nepel A., Barison A., Costa E.V. (2014). A New Source of (R)-Limonene and Rotundifolone from Leaves of Lippia Pedunculosa (Verbenaceae) and Their Trypanocidal Properties. Nat. Prod. Commun..

[32] Costa E.V., Dutra L.M., Nogueira P.C.D.L., Moraes V.R.D.S., Salvador M.J., Ribeiro L.H.G., Gadelha F.R. (2012). Essential Oil from the Leaves of Annona Vepretorum: Chemical Composition and Bioactivity. Nat. Prod. Commun..

[33] Bay M., Souza de Oliveira J.V., Sales Junior P.A., Fonseca Murta S.M., Rogério dos Santos A., dos Santos Bastos I., Puccinelli Orlandi P., Teixeira de Sousa Junior P. (2019). In Vitro Trypanocidal and Antibacterial Activities of Essential Oils from Four Species of the Family Annonaceae. Chem. Biodivers..

[34] González-Coloma A., Sainz P., Andrés M.F., Martínez-Díaz R.A., Bailén M., Navarro-Rocha J., Díaz C.E. (2019). Chemical Composition and Biological Activities of Artemisia Pedemontana Subsp. Assoana Essential Oils and Hydrolate. Biomolecules.

[35] Gutiérrez Y., Montes R., Scull R., Sánchez A., Cos P., Monzote L., Setzer W.N. (2016). Chemodiversity Associated with Cytotoxicity and Antimicrobial Activity of *Piper Aduncum* Var. Ossanum. Chem. Biodivers..

[36] Kian D., Lancheros C.A.C., Assolini J.P., Arakawa N.S., Veiga-Júnior V.F., Nakamura C.V., Pinge-Filho P., Conchon-Costa I., Pavanelli W.R., Yamada-Ogatta S.F. (2018). Trypanocidal Activity of Copaiba Oil and Kaurenoic Acid Does Not Depend on Macrophage Killing Machinery. Biomed. Pharmacother..

[37] Moreno É.M., Leal S.M., Stashenko E.E., García L.T. (2018). Induction of Programmed Cell Death in Trypanosoma Cruzi by Lippia Alba Essential Oils and Their Major and Synergistic Terpenes (Citral, Limonene and Caryophyllene Oxide). BMC Complement. Altern. Med..

[38] Pereira P.S., Maia A.J., Duarte A.E., Oliveira-Tintino C.D.M., Tintino S.R., Barros L.M., Vega-Gomez M.C., Rolón M., Coronel C., Coutinho H.D.M. (2018). Cytotoxic and Anti-Kinetoplastid Potential of the Essential Oil of Alpinia Speciosa, K. Schum. Food Chem. Toxicol..

[39] Zanusso Junior G., Massago M., Kian D., Toledo M.J.d.O. (2018). Efficacy of Essential Oil of Syzygium Aromaticum Alone and in Combination with Benznidazole on Murine Oral Infection with Trypanosoma Cruzi IV. Exp. Parasitol..

[40] Oliveira de Souza L.I., Bezzera-Silva P.C., do Amaral Ferraz Navarro D.M., da Silva A.G., dos Santos Correia M.T., da Silva M.V., de Figueiredo R.C.B.Q. (2017). The Chemical Composition and Trypanocidal Activity of Volatile Oils from Brazilian Caatinga Plants. Biomed. Pharmacother..

[41] Estevam E.B.B., De Deus I.P.B., Da Silva V.P., Da Silva E.A.J., Alves C.C.F., Alves J.M., Cazal C.M., Magalhães L.G., Pagotti M.C., Esperandim V.R. (2017). In Vitro Antiparasitic Activity and Chemical Composition of the Essential Oil from Protium Ovatum Leaves (Burceraceae). An. Acad. Bras. Cienc..

[42] Tasdemir D., Kaiser M., Demirci B., Demirci F., Baser K. (2019). Antiprotozoal Activity of Turkish Origanum Onites Essential Oil and Its Components. Molecules.

[43] Gutiérrez Y.I., Scull R., Villa A., Satyal P., Cos P., Monzote L., Setzer W.N. (2019). Chemical Composition, Antimicrobial and Antiparasitic Screening of the Essential Oil from Phania Matricarioides (Spreng.) Griseb. Molecules.

[44] Kpoviessi S., Bero J., Agbani P., Gbaguidi F., Kpadonou-Kpoviessi B., Sinsin B., Accrombessi G., Frédérich M., Moudachirou M., Quetin-Leclercq J. (2014). Chemical Composition, Cytotoxicity and in Vitro Antitrypanosomal and Antiplasmodial Activity of the Essential Oils of Four Cymbopogon Species from Benin. J. Ethnopharmacol..

[45] Saeidnia S., Gohari A.R., Hadjiakhoondi A., Shafiee A. (2007). Bioactive Compounds of the Volatile Oil of Dracocephalum Kotschyi. Zeitschrift fur Naturforsch. Sect. C J. Biosci..

[46] Nibret E., Wink M. (2010). Trypanocidal and Antileukaemic Effects of the Essential Oils of Hagenia Abyssinica, Leonotis Ocymifolia, Moringa Stenopetala, and Their Main Individual Constituents. Phytomedicine.

[47] Bero J., Beaufay C., Hannaert V., Hérent M.F., Michels P.A., Quetin-Leclercq J. (2013). Antitrypanosomal Compounds from the Essential Oil and Extracts of Keetia Leucantha Leaves with Inhibitor Activity on Trypanosoma Brucei Glyceraldehyde-3-Phosphate Dehydrogenase. Phytomedicine.

[48] Kpadonou Kpoviessi B.G.H., Kpoviessi S.D.S., Yayi Ladekan E., Gbaguidi F., Frédérich M., Moudachirou M., Quetin-Leclercq J., Accrombessi G.C., Bero J. (2014). In Vitro Antitrypanosomal and Antiplasmodial Activities of Crude Extracts and Essential Oils of Ocimum Gratissimum Linn from Benin and Influence of Vegetative Stage. J. Ethnopharmacol..

[49] Cheikh-Ali Z., Adiko M., Bouttier S., Bories C., Okpekon T., Poupon E., Champy P. (2011). Composition, and Antimicrobial and Remarkable Antiprotozoal Activities of the Essential Oil of Rhizomes of Aframomum Sceptrum, K. Schum. (Zingiberaceae). Chem. Biodivers..

[50] Mulyaningsih S., Youns M., El-Readi M.Z., Ashour M.L., Nibret E., Sporer F., Herrmann F., Reichling J., Wink M. (2010). Biological Activity of the Essential Oil of Kadsura Longipedunculata (Schisandraceae) and Its Major Components. J. Pharm. Pharmacol..

[51] Rolón M., Vega C., Escario J.A., Gómez-Barrio A. (2006). Development of Resazurin Microtiter Assay for Drug Sensibility Testing of Trypanosoma Cruzi Epimastigotes. Parasitol. Res..

[52] De Sousa D.P., Lima T.C., Steverding D. (2016). Evaluation of Antiparasitic Activity of Mentha Crispa Essential Oil, Its Major Constituent Rotundifolone and Analogues against Trypanosoma Brucei. Planta Med..

[53] Petrelli R., Orsomando G., Sorci L., Maggi F., Ranjbarian F., Biapa Nya P.C., Petrelli D., Vitali L.A., Lupidi G., Quassinti L. (2016). Biological Activities of the Essential Oil from Erigeron Floribundus. Molecules.

[54] Sobeh M., Braun M.S., Krstin S., Youssef F.S., Ashour M.L., Wink M. (2016). Chemical Profiling of the Essential Oils of Syzygium Aqueum, Syzygium Samarangense and Eugenia Uniflora and Their Discrimination Using Chemometric Analysis. Chem. Biodivers..

[55] Petrelli R., Ranjbarian F., Dall’Acqua S., Papa F., Iannarelli R., Ngahang Kamte S.L., Vittori S., Benelli G., Maggi F., Hofer A. (2017). An Overlooked Horticultural Crop, Smyrnium Olusatrum, as a Potential Source of Compounds Effective against African Trypanosomiasis. Parasitol. Int..

[56] Costa S., Cavadas C., Cavaleiro C., Salgueiro L., do Céu Sousa M. (2018). In Vitro Susceptibility of Trypanosoma Brucei Brucei to Selected Essential Oils and Their Major Components. Exp. Parasitol..

[57] Ngahang Kamte S.L., Ranjbarian F., Cianfaglione K., Sut S., Dall’Acqua S., Bruno M., Afshar F.H., Iannarelli R., Benelli G., Cappellacci L. (2018). Identification of Highly Effective Antitrypanosomal Compounds in Essential Oils from the Apiaceae Family. Ecotoxicol. Environ. Saf..

[58] Kamte S.L.N., Ranjbarian F., Campagnaro G.D., Nya P.C.B., Mbuntcha H., Woguem V., Womeni H.M., Tapondjou L.A., Giordani C., Barboni L. (2017). Trypanosoma Brucei Inhibition by Essential Oils from Medicinal and Aromatic Plants Traditionally Used in Cameroon (Azadirachta Indica, Aframomum Melegueta, Aframomum Daniellii, Clausena Anisata, Dichrostachys Cinerea and Echinops Giganteus). Int. J. Environ. Res. Public Health.

[59] Hoet S., Stévigny C., Hérent M.F., Quetin-Leclercq J. (2006). Antitrypanosomal Compounds from the Leaf Essential Oil of Strychnos Spinosa. Planta Med..

[60] Le T., Beaufay C., Nghiem D., Pham T., Mingeot-Leclercq M., Quetin-Leclercq J. (2019). Evaluation of the Anti-Trypanosomal Activity of Vietnamese Essential Oils, with Emphasis on Curcuma Longa, L. and Its Components. Molecules.

[61] Do Carmo G.M., Baldissera M.D., Vaucher R.A., Rech V.C., Oliveira C.B., Sagrillo M.R., Boligon A.A., Athayde M.L., Alves M.P., França R.T. (2015). Effect of the Treatment with Achyrocline Satureioides (Free and Nanocapsules Essential Oil) and Diminazene Aceturate on Hematological and Biochemical Parameters in Rats Infected by Trypanosoma Evansi. Exp. Parasitol..

[62] Baldissera M.D., Oliveira C.B., Zimmermann C.E.P., Boligon A.A., Athayde M.L., Bolzan L.P., Vaucher R.d.A., Santurio J.M., Sagrillo M.R., da Silva A.S. (2014). In Vitro Trypanocidal Activity of Macela (Achyrocline Satureioides) Extracts against Trypanosoma Evansi. Korean, J. Parasitol..

[63] Giongo J.L., Vaucher R.A., Da Silva A.S., Oliveira C.B., de Mattos C.B., Baldissera M.D., Sagrillo M.R., Monteiro S.G., Custódio D.L., Souza de Matos M. (2017). Trypanocidal Activity of the Compounds Present in Aniba Canelilla Oil against Trypanosoma Evansi and Its Effects on Viability of Lymphocytes. Microb. Pathog..

[64] Baldissera M.D., D. Silva A.S., Oliveira C.B., Zimmermann C.E.P., Vaucher R.A., Santos R.C.V., Rech V.C., Tonin A.A., Giongo J.L., Mattos C.B. (2013). Trypanocidal Activity of the Essential Oils in Their Conventional and Nanoemulsion Forms: In Vitro Tests. Exp. Parasitol..

[65] Baldissera M.D., de Freitas Souza C., Mourão R.H.V., da Silva L.V.F., Monteiro S.G. (2017). Trypanocidal Action of Lippia Alba and Lippia Origanoides Essential Oils against Trypanosoma Evansi in Vitro and in Vivo Used Mice as Experimental Model. J. Parasit. Dis..

[66] Oliveira M.S., Almeida M.M., Salazar M.L.A., Pires F.C., Bezerra F.W., Cunha V.M., Cordeiro R.M., Urbina G.R., Silva M.P., Silva A.P.S., El-Shemy H.A. (2018). Potential of Medicinal Use of Essential Oils from Aromatic Plants. Potential of Essential Oils.

[67] Yang N.J., Hinner M. (2015). Getting Across the Cell Membrane: An Overview for Small Molecules, Peptides, and Proteins. Methods Mol. Biol..

[68] Yoon J., Ben-Ami H.C., Hong Y.S., Park S., Strong L.L.R., Bowman J., Geng C., Baek K., Minke B., Pak W.L. (2000). Novel Mechanism of Massive Photoreceptor Degeneration Caused by Mutations in the Trp Gene of Drosophila. J. Neurosci..

[69] Giorgio V., Guo L., Bassot C., Petronilli V., Bernardi P. (2018). Calcium and Regulation of the Mitochondrial Permeability Transition. Cell Calcium.

[70] Menna-Barreto R., Pons A., Pinto A., Diaz J., Soares M., De Castro S. (2005). Effect of a Beta-Lapachone-Derived Naphthoimidazole on Trypanosoma cruzi: Identification of Target Organelles. J. Antimicrob. Chemother..

[71] Elmore S. (2007). Apoptosis: A Review of Programmed Cell Death. Toxicol. Pathol..

[72] Grivicich I., Regner A., da Rocha A.B. (2007). Morte Celular Por Apoptose. Rev. Bras. Cancrol..

